# Trunk muscle co-activation using functional electrical stimulation modifies center of pressure fluctuations during quiet sitting by increasing trunk stiffness

**DOI:** 10.1186/s12984-015-0091-8

**Published:** 2015-11-10

**Authors:** Matija Milosevic, Kei Masani, Noel Wu, Kristiina M. V. McConville, Milos R. Popovic

**Affiliations:** Institute of Biomaterials and Biomedical Engineering, University of Toronto, 164 College Street, Toronto, ON M5S 3G9 Canada; Rehabilitation Engineering Laboratory, Lyndhurst Centre, Toronto Rehabilitation Institute – University Health Network, 520 Sutherland Drive, Toronto, ON M4G 3V9 Canada; Department of Electrical and Computer Engineering, Ryerson University, 350 Victoria Street, Toronto, ON M5B 2K3 Canada

**Keywords:** Trunk, Posturography, Quiet sitting, Functional electrical stimulation (FES), Stiffness, Inverted pendulum model

## Abstract

**Background:**

The purpose of this study was to examine the impact of functional electrical stimulation (FES) induced co-activation of trunk muscles during quiet sitting. We hypothesized that FES applied to the trunk muscles will increase trunk stiffness. The objectives of this study were to: 1) compare the center of pressure (COP) fluctuations during unsupported and FES-assisted quiet sitting - an experimental study and; 2) investigate how FES influences sitting balance - an analytical (simulation) study.

**Methods:**

The experimental study involved 15 able-bodied individuals who were seated on an instrumented chair. During the experiment, COP of the body projected on the seating surface was calculated to compare sitting stability of participants during unsupported and FES-assisted quiet sitting. The analytical (simulation) study examined dynamics of quiet sitting using an inverted pendulum model, representing the body, and a proportional-derivative (PD) controller, representing the central nervous system control. This model was used to analyze the relationship between increased trunk stiffness and COP fluctuations.

**Results:**

In the experimental study, the COP fluctuations showed that: i) the mean velocity, mean frequency and the power frequency were higher during FES-assisted sitting; ii) the frequency dispersion for anterior-posterior fluctuations was smaller during FES-assisted sitting; and iii) the mean distance, range and centroidal frequency did not change during FES-assisted sitting. The analytical (simulation) study showed that increased mechanical stiffness of the trunk had the same effect on COP fluctuations as the FES.

**Conclusions:**

The results of this study suggest that FES applied to the key trunk muscles increases the speed of the COP fluctuations by increasing the trunk stiffness during quiet sitting.

## Background

The human spine is inherently unstable and trunk musculature, which surrounds the spine, is primarily responsible for maintaining its stability against multidirectional external forces [1–4]. During quiet sitting, weak tonic activation of the trunk muscles (1–3 % of the maximum voluntary contraction for abdominal muscles, and 4–6 % for back muscles) provides sufficient multidirectional trunk stiffness to ensure stable quiet sitting [2]. Neurological injuries, such as spinal cord injury (SCI) and traumatic brain injury, impact sitting balance. They often result in neuromuscular deficits that cause postural instability [5] and the inability to effectively compensate for external perturbations [6–9].

Functional electrical stimulation (FES) can generate muscle contractions by delivering short electric pulses between two electrodes placed on the surface of the skin over the muscle nerve [10]. Continuous co-activation of trunk muscles with FES was previously shown to improve clinical measures of static balance by correcting spinal alignment [8] and dynamic balance during forward reaching [7] in people with SCI. It was also shown that stimulation of the trunk muscles with FES can produce sufficient trunk muscle contractions to stabilize up to 45 % of body weight during sitting balance perturbations [6]. Triolo and colleagues [7, 8] showed that FES of the trunk muscles can cause postural improvements during sitting balance and they assumed that the improvements were caused by increased trunk stiffness. Previous studies have demonstrated that voluntarily co-activation of trunk muscles increases trunk stiffness [11, 12]. Therefore, it is logical that trunk muscle co-activation induced by FES, could also increase trunk stiffness. Moreover, activation of trunk muscles using FES was shown to increase multidirectional trunk stiffness during perturbed sitting balance [9].

To date, no study has investigated how FES applied to the trunk muscles influences postural sway and trunk stiffness during quiet sitting. Postural control during quiet sitting [3, 5, 13] and standing [14–16], which can be evaluated using the center of pressure (COP) sway fluctuations, has been utilized to characterize the balance control of these two biomechanical systems. To understand the effects of trunk stability on the sitting balance control system, Reeves et al. [3] investigated the COP fluctuations during quiet sitting on an unstable surface. Their findings suggest that additional voluntary trunk muscle co-activations, which are believed to increase trunk stiffness, increased the COP velocity. Using COP sway measures during quiet sitting, it was also shown that individuals with SCI have compromised control of the trunk, which is caused by their neuromuscular impairment [5]. Further, COP sway measures when combined with computational simulations could be used to quantitatively analyze the underlying postural control mechanisms, including the contribution of stiffness and damping during balance control [15].

We hypothesized that co-activating the trunk muscles with FES will modify sitting balance by increasing trunk stiffness. The objectives of this study were to: 1) compare the COP fluctuations during unsupported quiet sitting to FES-assisted quiet sitting - an experimental study and; 2) investigate how FES influences sitting balance - an analytical (simulation) study.

## Methods

### Experimental study

#### Participants

Fifteen male able-bodied individuals (age 26.7 ± 4.6 years; weight 72.5 ± 8.1 kg; height 175.7 ± 6.7 cm) participated in this study. None of the participants had a history of neurological and sensory impairments, and musculoskeletal injury that could compromise their sitting balance. All participants gave written informed consent in accordance with the principles of the Declaration of Helsinki. The experimental procedures were approved by the local institutional ethics committee.

#### Study protocol

**Fig. 1:**
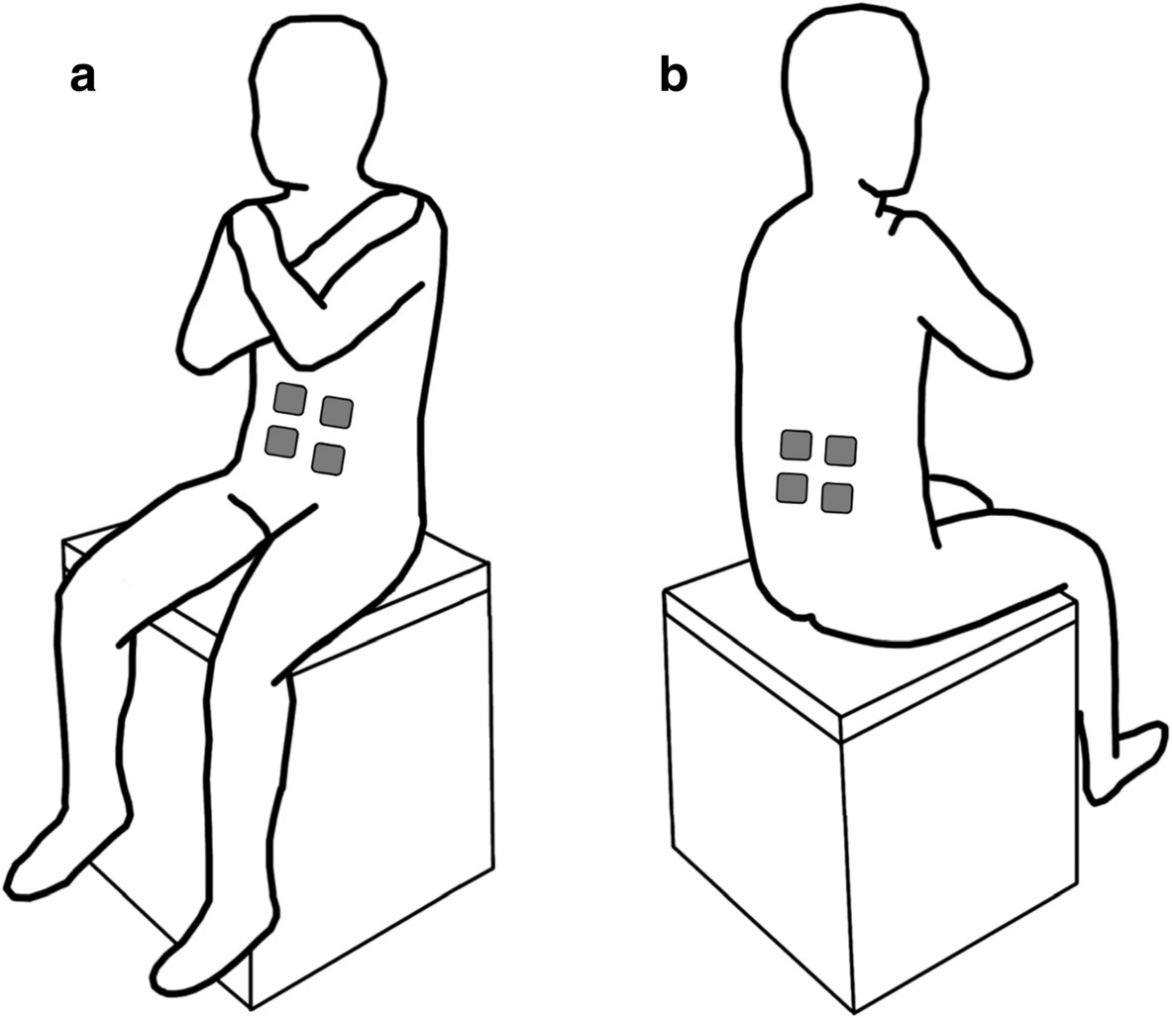
Experimental setup showing participant’s posture on a chair without back support during sitting balance assessments. The force plate was positioned on the seat surface, under the buttocks, to capture trunk sway, while the participant’s feet were not supported on the ground and the participants had their arms crossed on their chest. The figure also shows the: **a** front view of the participant illustrating the approximate location of the FES electrodes on the rectus abdominis (RA) muscle and; **b** back view of the participant illustrating the approximate location of the of the FES electrodes on the lumbar erector spinae (L3) muscle. The RA and L3 muscles were stimulated bilaterally and were activated simultaneously to generate co-activations

Participants were asked to maintain an upright sitting posture on a height-adjustable instrumented chair without back support, such that their feet were not supported on the ground and with their arms crossed on their chest (Fig. 1). Participants maintained quiet sitting posture during: a) unsupported sitting; and b) FES-assisted sitting conditions. The order of the two sitting conditions was randomized between participants. Before data collection, participants were given an opportunity to become familiarized with FES-assisted sitting. For each condition, data was collected over two, 30 s trials.

#### Functional electrical stimulation (FES)

During FES-assisted sitting, a portable FES system Complex Motion (Compex, Switzerland [10]) was used to deliver transcutaneous electrical stimulation to the trunk muscles by applying rectangular, biphasic, asymmetric charge balanced stimulation pulses with a 300 μsec pulse duration and 40 Hz frequency via self-adhesive gel electrodes (5 × 5 cm). Stimulation electrodes were placed on rectus abdominis (RA) and lumbar portions of the erector spinae (L3) muscles bilaterally, and were activated simultaneously to generate co-activations (Fig. 1). These muscles were chosen because they contribute significantly to trunk stability during sitting balance perturbations [4]. The stimulation intensity for each muscle was determined by gradually increasing the stimulation amplitude with 1 mA increments until the experimenter identified the motor threshold by checking for palpable contractions. The stimulation intensity was then set to twice the motor threshold, or the highest tolerable amplitude, which was higher than the motor threshold but less than twice the motor threshold. Trunk flexors and extensors were symmetrically activated to avoid trunk bending. The average stimulation amplitude was 20.3 ± 3.8 mA for the RA and 24.6 ± 7.4 mA for the L3 muscles.

#### Center of pressure (COP)

Ground-reaction forces were recorded using a force plate AccuSway^Plus^ (Advanced Mechanical Technology Inc., USA) positioned on the seat, under the buttocks of the participants (Fig. 1). Force plate signals were sampled at 500 Hz using a 12-bit data acquisition system NI 6071E (National Instruments, USA). COP fluctuations in anterior-posterior (AP) and medial-lateral (ML) directions were calculated from the recordings [14]. A low-pass filter with a cut-off frequency of 5 Hz was applied to all recordings [13, 14].

The time and frequency domain parameters were calculated to characterize the COP fluctuations as described in [14]. COP fluctuations were referenced by subtracting the mean position from each time series [14, 15]. Time-domain measures included: a) mean distance (MD), which represented the average distance from the origin travelled by the COP; b) mean velocity (MV), which was the average velocity of the COP time series; c) range (RANGE), which was the maximum distance between the two points on the COP path; and d) mean frequency (MFREQ), which was a measure that described the frequency of a sinusoidal osculation of the COP series derived from the ratio of the mean velocity to the mean distance. The frequency-domain parameters characterized the area or shape of the power spectral density of the COP series and were calculated in the frequency range from 0.15 to 5.0 Hz [5, 13, 14]. They included: a) centroidal frequency (CFREQ), which represented the central mass frequency; b) frequency dispersion (FREQD), which was a unit-less measure of the variability of the power spectral density; and c) 50 % power (P50), which included the frequencies below which 50 % of the total power of the spectral density was concentrated. All selected measures were chosen to accurately describe the COP fluctuations and have been used extensively in the literature [14, 15]. Statistical analysis was performed to compare all parameters during unsupported and FES-assisted quiet sitting using the Wilcoxon signed-ranks test. A non-parametric test was chosen because Shapiro-Wilk test has shown that not all selected measures were normally distributed. Significance level was set at *p* < 0.05.

### Analytical (simulation) study

#### Model

**Fig. 2:**
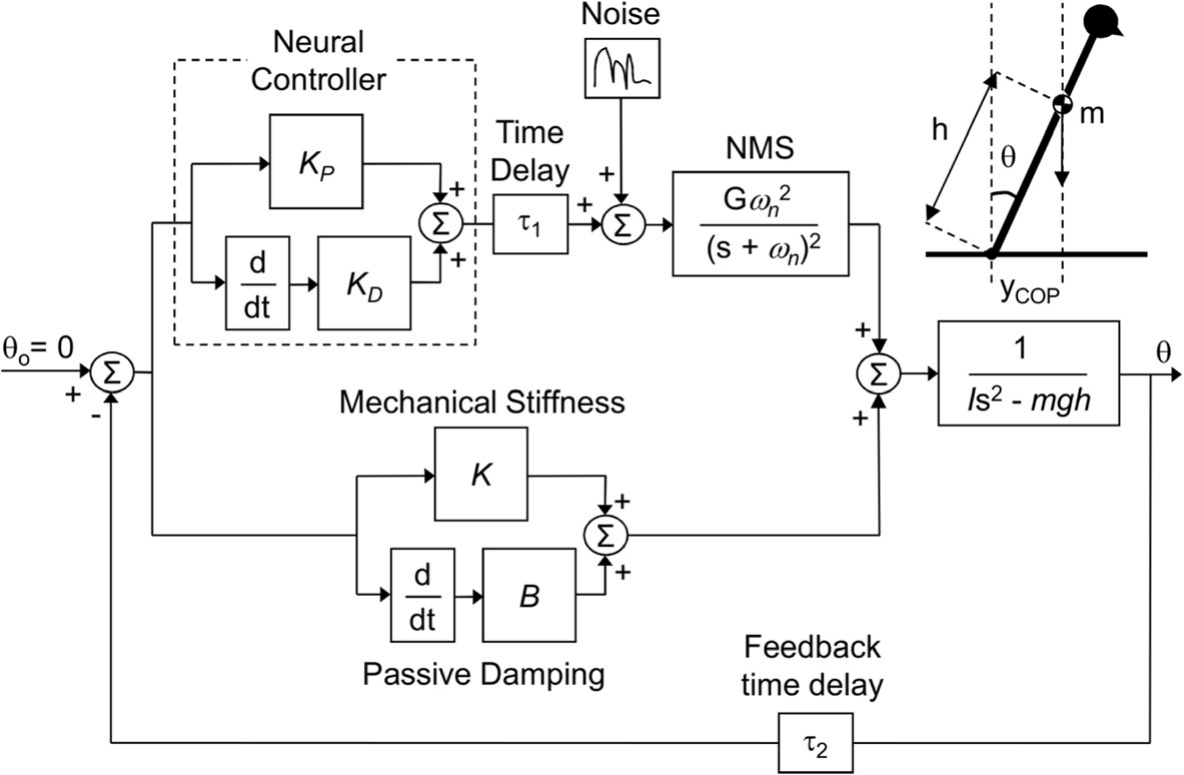
Block diagram of the model used in the simulation study. The feedback model included the neural controller with transmission delays (τ_1_, transmission time delay and τ_2_, feedback time delay) and the neuromusculoskeletal (NMS) torque-generation process, as well as mechanical stiffness (*K*) and passive damping (*B*) to control the inverted pendulum. The inverted pendulum was used to describe the mechanics of the quiet sitting. *m* is the moving mass, *h* is the height of center of mass (COM), and *I* is the moment of inertia of the inverted pendulum. *K*_*P*_ and *K*_*D*_, are proportional and derivative gains of the proportional-derivative (PD) controller, respectively, used to emulate the neural controller. An inverted pendulum model of quiet sitting is represented, where *y*_*COP*_ is the center of pressure (COP) position, *θ* is the sway angle, and g is the acceleration of gravity. Gaussian random noise was inserted into the system to drive the simulations

We conducted an analytical (simulation) study to investigate the mechanism of changes of COP fluctuations in AP direction, which were observed in the experiments described in Section 2.1. The simulation study was performed using Matlab and Simulink (ver. R2011b, MathWorks, Inc., USA). A feedback model of the control system during quiet sitting was developed using: i) an inverted pendulum model to describe the mechanics of the quiet sitting; ii) a proportional-derivative (PD) controller to represent the neural controller of the central nervous system that regulates balance of the trunk; iii) motor and sensory command transmission delays; iv) a neuromusculoskeletal (NMS) torque-generation process, which was modeled using a second order dynamic equation; and v) mechanical stiffness and passive damping of the trunk. All components of the model are shown in Fig. 2. A detailed description follows.

The size of the inverted pendulum was calculated using the average subjects’ body mass (M) and height (H), which was M = 72.5 kg and H = 1.76 m in this study. The moving mass (*m*), height of the center of mass (COM) (*h*), and the moment of inertia (*I*) of the moving part of the body (i.e., head, arms and trunk) with respect to the greater trochanter were estimated as: *m* = 0.678 M; *h* = 1.142(0.190H); and *I* = *mh*^2^, as described by Winter [17], to obtain *m* = 49.2 kg; *h* = 0.381 m; and *I* = 7.14 kg^.^m^2^. The neural control was modelled as the PD controller with a proportional gain (*K*_*P*_) and a derivative gain (*K*_*D*_) because it was shown that a PD controller can represent postural dynamics during both standing [15, 16, 18] and sitting balance [6]. A constant time delay was added to correspond to the motor (τ_1_) and sensory (τ_2_) transmission time delays of the central nervous system. Onset latencies of paraspinal muscles were shown to be 21.8 ms [19] and transmission time of approximately 25 ms was obtained empirically [20]. Also, transmission time between the motor cortex and the trunk muscles, obtained by motor evoked potentials, was shown to be between 15.2 and 17.6 ms in able-bodied individuals [21]. Our model used the transmission time delay of τ_1_ = 20 ms, and the feedback time delay of τ_2_ = 20 ms. The NMS torque-generation process, was modelled as a critically damped second-order system [18], where *ω*_*n*_ was the natural frequency of the second-order system. *T* = 1/*ω*_*n*_ corresponded to the twitch contraction time of the muscle (i.e. delay from the muscle contraction to the time force is generated). Thelen et al. [22] showed this time to be in the range between 111 and 218 ms for trunk muscles. Masani et al. [18] empirically derived the contraction times for the ankle muscles in the range between 121 and 192 ms. We chose the twitch contraction times to include *T* = 120, 170 and 220 ms, since it is known that NMS process affects the postural control mechanism [18]. The output of the neural controller delayed by the transmission time and the NMS system represented the active torque component. Mechanical properties of the system, including passive damping (*B*) and mechanical stiffness (*K*) were implemented separately, modeling the mechanical support structure, corresponding to the passive torque components. Gaussian random white noise was inserted into the system at the neural controller level, as shown in Fig. 2, to drive the simulations.

#### Simulation and analysis

The tested controller gain combinations included: 0 < *K*_*P*_ < 600 Nm/rad; 0 < *K*_*D*_ < 200 Nm^.^s/rad; and 0 < *K* < 300 Nm/rad, in increments of 20. The selected controller gain values were based on the experimentally derived gain values for sitting balance [20] to reflect the physiological system. Damping was implemented as: *B* = 1 Nm^.^s/rad and *B* = 6 Nm^.^s/rad, which is in the range of previous studies and since it was shown that it does not considerably affect the system dynamics [18]. Nyquist stability analysis was performed on the open-loop system to determine the gain combinations that stabilize the closed-loop system.

The gain combinations that stabilized the system were then used to simulate COP fluctuations. Ten trials of 30 s, for each stable gain combination, were simulated to produce the COP fluctuations. The simulation period and the number of trials were adapted to provide the reliable and robust results which capture the COP fluctuations [15, 16, 23]. Only the steady state fluctuations were analyzed to eliminate the COP fluctuations that are present during the first few seconds of the simulation, i.e. the transient phase. The obtained sway angle of the COM (*θ* ) which corresponds to the trunk angle, was used to calculate the anterior-posterior COP (i.e., *y*_*COP*_). As body sway during quiet sitting is small, the COM and COP can be approximated as: *y*_*COM*_ ≈ *h* ⋅ sin *θ* and ${y}_{\mathrm{COP}}\approx {y}_{COM}+\frac{I}{mgh}{\ddot{y}}_{COM}$ [23]. The same COP parameters as in the experimental study were then calculated using the simulated COP fluctuations. The relationship between each COP parameter and controller gain parameters were analyzed using partial correlation analysis with the COP parameters as a dependent variable and controller gains as independent variables. The linear relationship between each COP parameter and the mechanical stiffness gain *K* was evaluated using partial correlation coefficient. Significance level was set at *p* < 0.05.

## Results

### Experimental study: Effects of FES on sitting balance

**Fig. 3:**
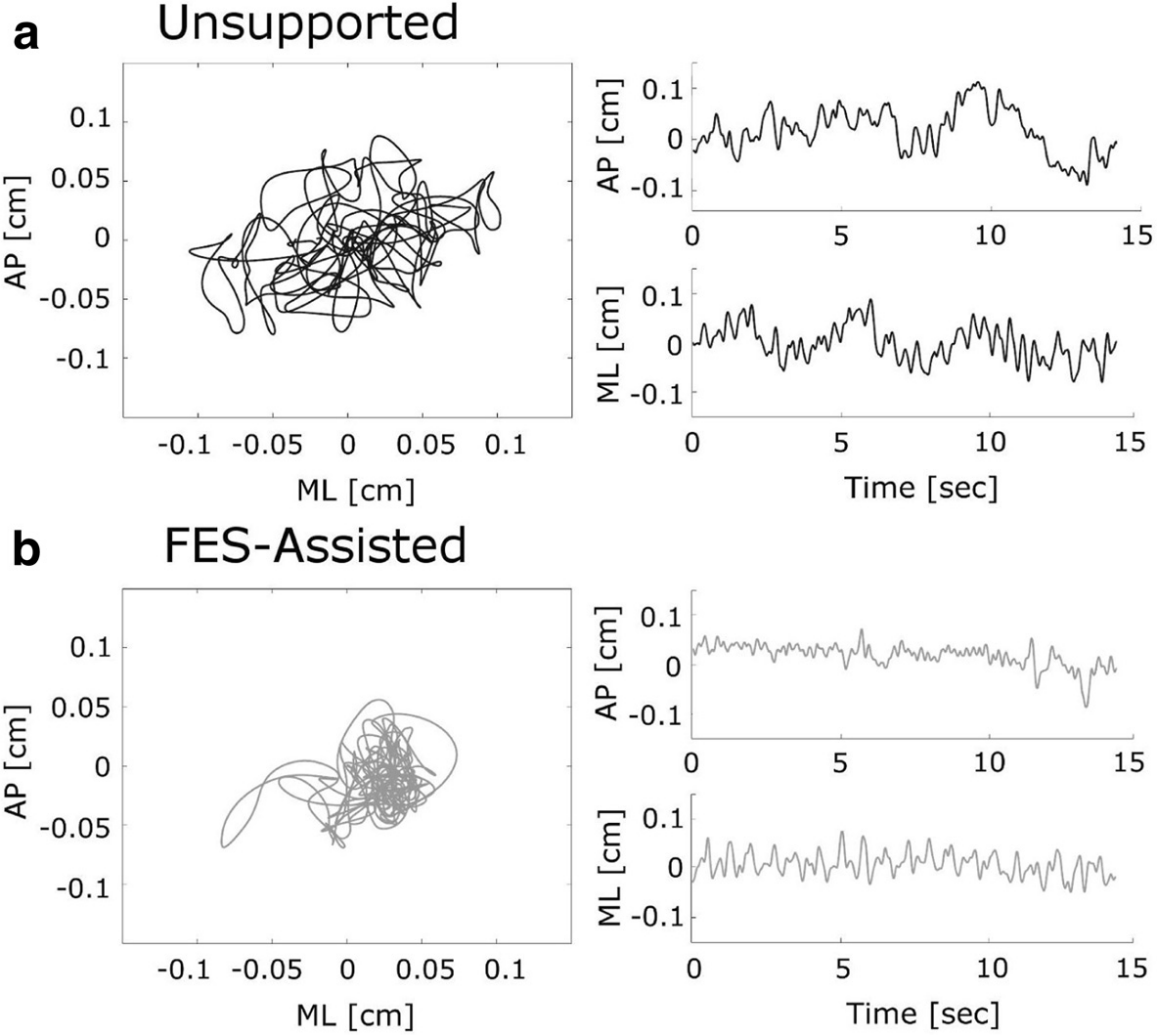
Example of the experimentally obtained center of pressure (COP) fluctuations during: **a** Unsupported quiet sitting and **b** FES-assisted quiet sitting for one participant. AP represents anterior-posterior and ML medial-lateral sway direction. The planar representations (*left*) show spatial fluctuations of the combined AP and ML sway. Time series plots (*right*) show the corresponding AP and ML postural sway time series separately. Note that only a representative 15 s of data is shown to describe the postural sway behaviour

The representative plots of one participant, shown in Fig. 3, illustrate that the participant seemed to sway faster and slightly less during FES-assisted sitting, compared to unsupported sitting. The obtained COP parameters are presented in Table 1. Comparison of time-domain parameters showed that the mean distance (MD) and the range (RANGE) tend to decrease with FES but the results were not statistically different. The mean velocity (MV) was significantly higher in the FES condition for anterior-posterior direction and the mean frequency (MFREQ) was significantly higher in the FES condition for both anterior-posterior and medio-lateral directions. For frequency domain parameters, frequency dispersion (FREQD) was significantly smaller and the 50 % power (P50) frequency was significantly higher for the anterior-posterior direction during FES-assisted sitting.

**Table 1:** Analysis of the anterior-posterior (AP) and medial-lateral (ML) center of pressure (COP) fluctuation parameters. Shown are: mean distance (MD), mean velocity (MV), range (RANGE), mean frequency (MFREQ), centroidal frequency (CFREQ), frequency dispersion (FREQD) and 50 % power (P50) frequency. Results show the mean ± S.D. for each COP fluctuation parameter and compare unsupported and FES-assisted sitting in 15 (*n* = 15) able-bodied individuals

Measures		Unsupported sitting	FES-Assisted sitting	Wilcoxon signed-ranks test
MD (mm)	AP	0.61 ± 0.26	0.54 ± 0.24	
	ML	0.52 ± 0.26	0.46 ± 0.28	
MV (mm/s)	AP	2.80 ± 0.41	3.04 ± 0.61	*
	ML	2.02 ± 0.54	2.30 ± 0.77	
RANGE (mm)	AP	0.40 ± 0.18	0.37 ± 0.14	
	ML	0.36 ± 0.23	0.33 ± 0.14	
MFREQ (Hz)	AP	0.97 ± 0.30	1.13 ± 0.35	*
	ML	0.82 ± 0.22	1.01 ± 0.27	*
CFREQ (Hz)	AP	1.72 ± 0.23	1.82 ± 0.25	
	ML	1.71 ± 0.19	1.67 ± 0.27	
FREQD (-)	AP	0.59 ± 0.05	0.56 ± 0.03	**
	ML	0.56 ± 0.05	0.54 ± 0.06	
P50 (Hz)	AP	0.50 ± 0.10	0.55 ± 0.13	*
	ML	0.48 ± 0.12	0.55 ± 0.14	

*p < 0.05; **p < 0.01

### Simulation study: Effects of increased stiffness on COP fluctuations

**Fig. 4:**
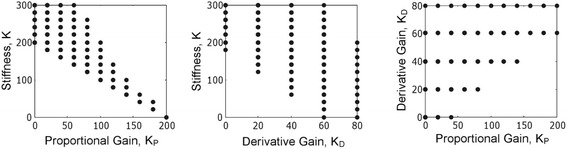
The gain combinations that stabilized the model used in the simulation study. *K*_*P*_ is the proportional gain, *K*_*D*_ is the derivative gain of the proportional-derivative (PD) controller used to emulate the neural control, and *K* is the mechanical stiffness contribution. The figure shows the relationship between the parameters

Figure 4 shows the stable gain combinations for the simulation study, which were selected via the Nyquist stability analysis. Minimum stiffness of *K* = 180 Nm/rad was sufficient to stabilize the system without the need for a neural controller. It can also be observed that only very high proportional and derivative gains, i.e. *K*_*P*_ = 200 Nm/rad and *K*_*D*_ = 60 Nm^.^s/rad, were able to stabilize the system without any passive damping and stiffness contributions (i.e., *B* = 0 Nm^.^s/rad and *K* = 0 Nm/rad).

Table 2 summarizes the results of the partial correlations analysis. *K* was positively correlated with mean velocity, mean frequency and 50 % power (MV, MFREQ and P50) and negatively correlated with frequency dispersion (FREQD). There was no correlation between *K* and the mean distance (MD), range (RANGE) and centroidal frequency (CFREQ).

**Table 2:** Partial correlations between simulated center of pressure (COP) fluctuations and mechanical stiffness controller gains. Shown are the coefficients of correlation between each COP measurement and the mechanical stiffness gain which was varied as 0 < *K* < 300 Nm/rad, while controlling for the effect of proportional gain (*K*_*P*_) and derivative gain (*K*_*D*_). Included are the mean distance (MD), mean velocity (MV), range (RANGE), mean frequency (MFREQ), centroidal frequency (CFREQ), frequency dispersion (FREQD) and 50 % power (P50) frequency, obtained in the simulation study

	*Correlations*	
Measures	*K*	
MD	−0.035	
MV	0.283	**
RANGE	−0.096	
MFREQ	0.927	**
CFREQ	−0.034	
FREQD	−0.543	**
P50	0.422	**

**p < 0.01

## Discussions

### FES modifies sitting balance

Experimental results indicate that FES applied to the trunk muscles during quiet sitting makes the COP move faster (i.e. higher MV and P50), while it does not affect the amount of COP fluctuations (i.e. MD and RANGE). Results also showed that frequency dispersion (i.e. FREQD) was smaller during FES-assisted sitting, indicating that the frequency components of COP fluctuations became less variable with the application of FES (Table 1).

It has been previously shown that the COP velocity is higher in elderly people than in young people and that the amount of COP fluctuations is not different between the two populations during standing [14]. Prieto et al. [14] attributed these differences to stiffness in the lower limbs, which were observed in elderly people. Reeves et al. [3] reported that voluntary trunk muscle co-contraction during sitting, which was assumed to increase trunk stiffness, increased COP velocity. Using simulations, Maurer and Peterka [15] found correlations between frequency dispersion of the swaying object and the mechanical stiffness of that object. Consequently, our experimental results suggest that changes in COP fluctuation during FES-assisted sitting are due to increased trunk stiffness resulting from FES being applied to the trunk musculature. Stiffness is not necessarily a good balance control strategy for quiet sitting. However, increasing trunk stiffness prior to or during external perturbations can improve response to such perturbations [3, 6]. Contracting trunk muscles with FES after neurological injuries such as SCI could be useful for improving trunk control [5] and it could improve functional sitting balance [7, 8].

Our results also indicate that COP fluctuations during FES-assisted sitting primarily affected the anterior-posterior direction. This is likely because the muscles that we stimulated were trunk flexor and extensor muscles (i.e. rectus abdominis and lumbar erector spinae), which mainly control anterior-posterior stability [4]. These results suggest that if FES is applied to the trunk muscles, it could provide direction specific stiffness of the trunk, depending on which muscles are activated. The ability to selectively activate specific muscles may be a unique advantage of FES for control of trunk muscles during sitting balance.

### FES increases trunk stiffness

The analytical (simulation) study showed that increased mechanical stiffness *K* had the same effect on COP fluctuations as the application of FES on the trunk muscles. That is, in our simulation study, *K* was positively correlated with MV, MFREQ and P50, negatively correlated with FREQD, and not correlated at all with MD, RANGE and CFREQ. In the experimental study with FES-assisted sitting, FES applied to trunk muscles increased MV, MFREQ and P50, decreased FREQD and it did not change MD, RANGE and CFREQ. These results support the hypothesis that FES applied to the trunk muscles increased stiffness during sitting. This is similar to the findings of Lee et al. [11] who found that voluntary co-contraction of trunk muscles increases trunk stiffness. Maurer and Peterka [15] also examined the relationship between sway measures and model parameters during standing and showed that increased stiffness was generally associated with increased sway velocity, which also agrees with our experimental and simulation results.

The simulation study results showed that mechanical stiffness and proportional gain parameters (i.e. *K* and *K*_*P*_, respectively) stabilized the trunk and had an inverse relationship (Fig. 4). This is because the summation of *K* and *K*_*P*_ are approximately equal to the required stiffness of *mgh* = 184 Nm/rad, although it is possible for this value to be higher. Very large proportional gains can induce unstable posture but these gain combinations would be eliminated by the Nyquist stability criterion. Similar relationships between mechanical stiffness and neural controller gains were obtained by Masani et al. [18] in standing balance simulations. This relationship suggests that, in order to compensate for the reduced neural contributions (i.e. lower *K*_*P*_) in people with sitting postural instability due to neurological impairments, it is necessary to increase the mechanical stiffness (i.e. *K*). One possible way of achieving this is by applying FES to the muscles of interest (i.e. those that can increase stiffness along particular trunk axes).

### Implications for individuals with SCI

Trunk control is the dominant mechanism which is responsible for sitting balance impairment in individuals with SCI and it has been proposed that interventions for rehabilitation of the trunk are necessary to improve sitting in individuals with SCI [5]. Our study demonstrated the feasibility of co-activation of trunk muscles using FES to increase trunk stiffness during quiet sitting. It was previously suggested that such tonic activation of trunk muscles using FES could prevent immediate spine buckling and assist individuals with SCI during reaching [7, 8]. Moreover, it has also been shown that FES activation of the trunk muscles could also increase trunk stiffness during perturbed sitting [9], which can stabilize perturbations of up to 45 % of body weight in people with SCI [6]. Taken together, FES of the trunk muscles can be used to increase both tonic and phasic trunk stiffness, which is a desired strategy for maintaining sitting balance [16].

Stimulation of trunk muscles in individuals with SCI is more challenging. Depending on the level, severity and time since injury, trunk function after SCI could differ considerably from one individual to another. Moreover, muscle preservation, overall fitness, hydration, exact positioning of the electrodes and the muscle response to the stimulation all could affect the effectiveness on FES in individuals with SCI [24]. In co-contraction control it is very important to achieve balanced activations of the trunk muscle with FES to prevent trunk bending. Therefore, trunk function of individuals with SCI must be assessed carefully to design a customized FES stimulation protocol for each individual. In addition, the stimulation levels have to be adjusted frequently to account for changes such as fatigue of muscles due to FES [24].

### Limitations

Considering that it is difficult to manipulate only one element of postural control in the experiments, it is possible that there are other factors which could have also affected the results in addition to the increased trunk stiffness. Since FES is a noticeable stimulus and in order to maintain balanced activation of the trunk muscles and prevent trunk bending, it may be that able-bodied participants in our study voluntarily recruited other muscles in addition to those that were stimulated using FES, which may have also contributed to our findings. Increasing trunk stiffness may not be a desirable balance control strategy during quiet sitting as it could lead to muscle fatigue [7, 8]. However, increasing trunk stiffness can improve sitting in people with SCI [7, 8] and during external perturbations aimed at disrupting balance [6].

## Conclusions

Our experimental study results showed that FES applied to the trunk muscles modified the COP fluctuation during quiet sitting. Simulations that were performed as part of this study suggested that FES of the trunk muscles increased trunk stiffness. Previous models of sitting balance with FES provided some evidence in support of the idea that activation of trunk muscles using FES increased trunk stiffness. Our experimental and simulation results provided additional indication that co-activation of trunk muscles using FES indeed increased trunk stiffness during quiet sitting. Since FES can activate muscles in individuals with upper motor neuron deficit, such as people with SCI, it may be a viable strategy to apply FES on the trunk muscles to improve their sitting balance. As such, an FES intervention would be used to increase trunk stiffness and improve balance during quiet sitting. Since this was a preliminary study with able-bodied individuals, further experiments are required to fully confirm the effectiveness of FES to improve quiet sitting balance and capture the impacts of FES assistive technology in individuals with SCI.

## Abbreviations

FES: Functional electrical stimulation
COP: Center of pressure
PD: Proportional-derivative
SCI: Spinal cord injury
RA: Rectus abdominis
L3: Lumbar portions of the erector spinae
AP: Anterior-posterior
ML: Medial-lateral
MD: Mean distance
MV: Mean velocity
RANGE: Range
MFREQ: Mean frequency
CFREQ: Centroidal frequency
FREQD: Frequency dispersion
P50: 50 % power
NMS: Neuromusculoskeletal
M: Body mass
B: Body height
*m*: Moving mass
COM: Center of mass
*h*: Height of the COM
*I*: Moment of inertia
*K*_*P*_: Proportional gain
*K*_*D*_: Derivative gain
τ_1_: Motor transmission time delay
τ_2_: Sensory transmission time delay
*B*: Passive damping
*K*: Mechanical stiffness
*θ*: Angle of the COM
*y*_*COP*_: Anterior-posterior COP
